# Spectral CT in the Demonstration of the Pancreatic Arteries and Their Branches: A Comparison With Conventional CT

**DOI:** 10.1097/MD.0000000000002823

**Published:** 2016-02-18

**Authors:** Yan-Jie Shi, Xiao-Peng Zhang, Ying-Shi Sun, Li-Ping Qi, Ying Li, Hai-Bin Zhu, Xiao-Ting Li, Xiao-Yan Zhang

**Affiliations:** From the Key Laboratory of Carcinogenesis and Translational Research (Ministry of Education), Department of Radiology, Peking University Cancer Hospital & Institute, Beijing, China.

## Abstract

The aim of this study was to investigate the performance of monochromatic images of spectral computed tomographic (CT) in the visualization of the pancreatic arteries compared with polychromatic CT images.

We conducted a case–control study in a group of 26 consecutive patients with monochromatic CT and contrasted the results against a control group of 26 consecutive patients with polychromatic CT. The CNR (contrast-to-noise ratio), SIR (signal intensity ratio), SNR (signal to noise ratio), and image noise were measured. A 5-score classification system was used to evaluate the branch order of pancreatic arteries. The course of pancreatic arteries was compared.

Compared with polychromatic images, the CNR, SIR, and SNR obtained by monochromatic images were increased by 64.74%, 23.99%, and 39.50%. Branch visualization of PSPDA (posterior superior pancreaticoduodenal artery), ASPDA (anterior superior pancreaticoduodenal artery), and DPA (dorsal pancreatic artery) was better at monochromatic images than at polychromatic images. The display rate was significantly better in monochromatic images for the second and third segments of PSPDA, total course of ASPDA, and artery of uncinate process.

Compared with polychromatic images, monochromatic images can improve the visualization of pancreatic arteries.

## INTRODUCTION

Understanding the normal pancreatic arteries and their branches is especially important to the surgeons because these structures are vulnerable to injury during pancreatic surgery. In addition, the surgical technique of duodenum-preserving total head resection is to avoid resection of the duodenum and the extrahepatic biliary ducts for inflammatory and cystic neoplasitc disease of the pancreas head and uncinate process. Preservation of the posterior vessels of pancreatic head is the essential step to avoid the ischemia of the duodenum and biliary duct.^1,2^ Thus, it is essential to accurately assess the pancreatic arteries and their branches.

Multidetector computed tomography (MDCT) is one of the routinely used noninvasive modalities to display the pancreatic arteries. However, the polychromatic beam of a selected peak energy used in a conventional single-energy MDCT is more susceptible to beam hardening artifacts.^3^ Due to the relatively low resolution and contrast-to-noise ratio (CNR) of conventional computed tomography (CT) using a polychromatic X-ray beam, it is often difficult to display the small branches and total course of pancreatic arteries. Previous studies using helical CT are mainly focused on the origin and display rate of pancreatic arteries.^4,5^ Little had been reported about the branches and course of pancreatic arteries using MDCT.

Spectral CT is a newly developed technique that provided a potential solution for the aforementioned problems. A previous phantom study indicated that spectral CT images at approximately 70 keV can yield improved CNR without increasing the image noise.^6^ The recent studies mainly focused on the demonstration of enhanced vessels, such as coronary artery,^7,8^ pulmonary artery,^9^ portal vein,^10^ and abdominal arteries.^3,11^ These studies all indicated that spectral CT could yield improvements in the visualization of the enhanced vessels compared with the conventional CT images.

The purpose of this study was to investigate whether spectral CT with monochromatic images can improve the visualization of the branches and the course of pancreatic arteries compared with conventional CT with polychromatic images.

## METHODS

### Patients

This retrospective study was approved by our institutional review board, and a waiver of informed consent was remitted. The entry criteria for patients were the following: no history of pancreatic diseases; no liver, gallbladder, spleen, stomach malignancy; no history of heart and blood diseases; not received any therapy in abdomen. The exclusion criteria were the following: contraindications for the use of contrast and scan failure due to leakage of contrast, poor breath-holding.

From April to June 2013, 26 consecutive patients (12 male, 14 female; mean age, 55.8 ± 13.4 years) with spectral CT who met the entry criteria were retrospectively recruited from our clinics. Twenty-six consecutive patients (12 male, 14 female; mean age, 57.7 ± 11.2 years) examined with conventional polychromatic CT were also enrolled as control subjects in the study during the same period. The patients in the 2 groups were both patients with primary malignant tumor in pulmonary or pelvic cavity, and they received CT examination to evaluate whether there was abdominal metastasis.

### CT Protocol

All patients underwent precontrast and 2-phase (arterial and portal venous) enhanced CT examinations, with the Lightspeed 64 VCT (GE Medical Systems, Milwaukee, Wisconsin) or with the Discovery CT 750 HD scanner (GE Medical Systems, Milwaukee, Wisconsin).

The scanning parameters for spectral CT were as follows: spectral imaging scan mode with fast tube voltage switching between 80 and 140 kVp during a single rotation, tube currents of 600 mA, detector collimation 0.625 mm x 64; gantry rotation speed 0.6 s per rotation, and helical pitch of 0.984. An adaptive statistical iterative reconstruction (ASIR 30%) was used in spectral CT imaging.

The scanning parameters for conventional polychromatic CT were as follows: helical mode, tube voltage of 120 kVp, autoregulation of mA (200–400 mA) and noise index of 9, detector collimation 0.625 mm x 64; gantry rotation speed 0.6 s per rotation, helical pitch of 0.984.

All patients were scanned after at least 4 hours fast and in the supine position. The scan coverage was from diaphragmatic domes to lower sides of both kidneys. Patients were instructed to hold their breath, with tidal inspiration during scanning. After the plain scan, the nonionic contrast medium Iohexol (Omnipaque 300; GE Healthcare) at a dose of 1.5 mL/kg was injected at a rate of 4 mL/s through the median cubital vein.

An automatic bolus-tracking program was used to time the start of scanning in arterial phase after contrast material injection. A region-of-interest (ROI) cursor (0.8–2.0 cm^2^) was placed in the upper abdominal aorta. Arterial phase scanning was started as soon as possible after the enhancement threshold (aortic attenuation of 120 HU) was exceeded. Portal venous phase scan was carried out 60 seconds after the beginning of injection of the contrast medium.

### Image Postprocessing

The entire arterial phase CT imaging data were transferred to a workstation (Advantage Workstation 4.4; GE Healthcare). A radiologist analyzed and selected the optimal single energy (keV) level by applying optimal CNR software. On 70 keV, a manually defined 5 to 20 mm^2^ ROIs on SPA (splenic artery) were obtained; another ROI with the area of 15 mm^2^ on pancreatic body was measured in the same image. From the CNR plot, the optimal single energy (keV) level for generating the best CNR between the SPA and parenchyma of pancreatic body could be selected and saved. VR (volume rendering), MIP (maximum intensity projection), and CPR (curved planar reformation) were created for the both monochromatic and polychromatic images by a radiologist.

### Image Analysis

#### Quantitative Assessment

A radiologist (S.Y.J. with 7 years of experience in clinical CT) performed quantitative analysis in the optimal single energy level of monochromatic images and polychromatic images. Two ROIs with the area of 50 mm^2^ placed within the psoas in the same slice were measured. Two ROIs with the area of 15 mm^2^ on pancreas were measured in the same slice. Two manually defined 5 to 20 mm^2^ ROIs on SPA in the consecutive image were obtained; Images were magnified and care was taken to avoid the calcifications. The mean standard deviation (SD) within the psoas (which serves as a quantitative marker for image noise) and the mean CT number of the pancreas and the SPA were calculated.

CNR was a measure used to determine image quality. CNR was the ratio between the contrast enhancement of SPA and image noise. CNR, signal intensity ratio (SIR), and SNR were assessed using the following formula: CNR = (ROI 1 - ROI 2)/SD, SIR = ROI 1/ROI 2, SNR = ROI 1/SD, where ROI 1 was the CT number of the SPA, ROI 2 was the CT number of the pancreas, and SD was the image noise.

### Qualitative Assessment

**FIGURE 1:**
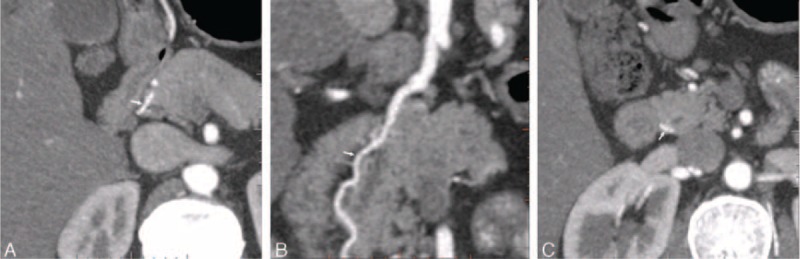
Visualization of the source of PSPDA with monochromatic images in a 36-year-old man. (A) The axial image showed the first segment with crossing anteriorly the common bile duct and running to the right of the common bile duct (arrow). (B) The coronal image showed the second segment with descending, parallel with the bile duct (arrow). (C) The axial image showed the third segment with crossing posteriorly the common bile duct and running to the left of the common bile duct (arrow).

**FIGURE 2:**
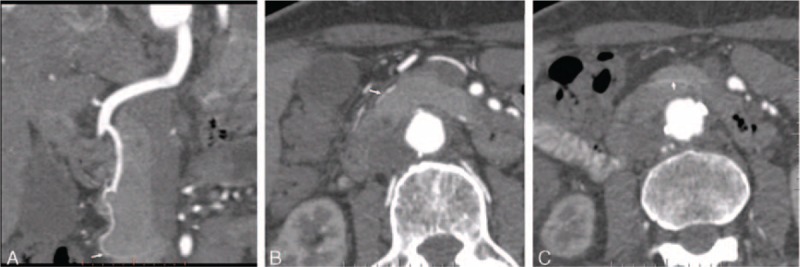
Visualization of the source of ASPDA with monochromatic images in a 76-year-old man. (A) CPR image showed the total source of ASPDA and the lower part of pancreatic head was noted (arrow). (B) The axial image showed that it run along the anterior and lateral surface of the pancreatic head (arrow). (C) The axial image showed that it went along the posterior surface of the uncinate process (arrow).

Two radiologists (S.Y.J. and Z.H.B, with 7 years of experience of abdominal CT, respectively) independently reviewed the arterial phase axial and reconstructed images. They were not made aware of any patient clinical information and CT imaging parameters. The visualization of the course of the pancreatic arteries was assessed with the following standard: According to the anatomic and DSA studies, the course of PSPDA were divided into 3 segments; the first segment was that it crossed anteriorly the common bile duct and run to the right of the bile tract; the second segment was that it descended, parallel with the common bile tract; the third segment was that it run to the left, crossing posteriorly the intrapancreatic portion of the common bile duct and ended anatomizing with the PIPDA^12^ (Figure 1). The ASPDA could run along the anterior and lateral surface of the head of pancreas and waving downward, parallel with the inner edge of the duodenum; the artery reached the lower part of the pancreatic head, turned backward, and went along the posterior surface of the uncinate process; we supposed that when this artery exceeded the lower part of the pancreatic head, total course of ASPDA was recorded^13,14^ (Figure 2). The artery of uncinate process originated from DPA, crossed the superior mesenteric vein posteriorly, and run along the left margin of the uncinate process.^15^ The 2 radiologists independently recorded whether segment of pancreatic artery could be displayed. Display rate was calculated by dividing the number of patients, whose segment of pancreatic arteries could be observed, by the total number of studied patients (26 patients). The display rate of segments of pancreatic arteries was evaluated by 2 radiologists independently and averaged for a mean value.

**FIGURE 3:**
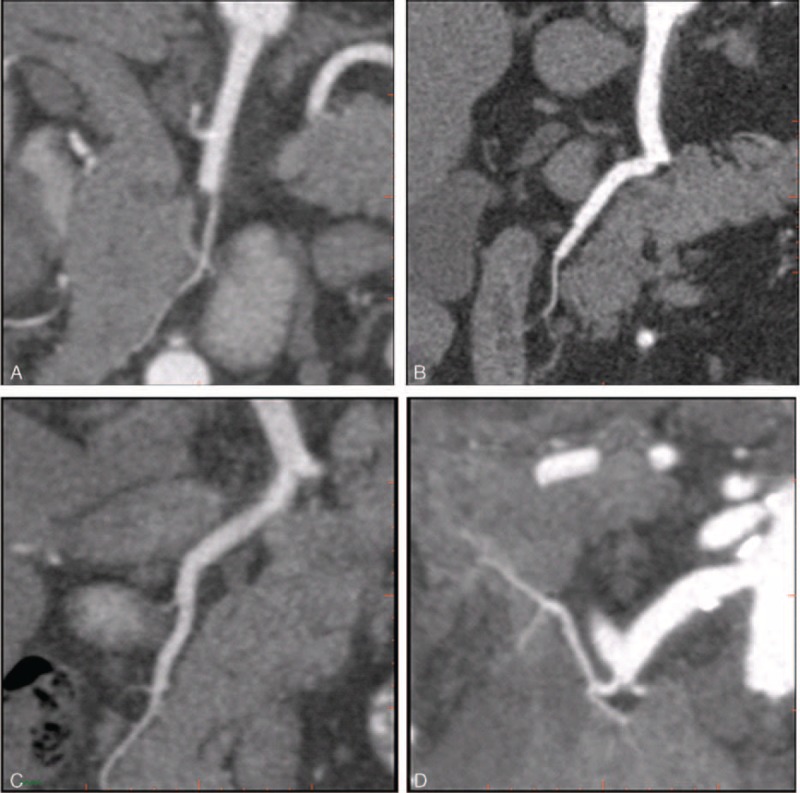
The visualization of the branch order of the pancreatic arteries with CPR images acquired by using spectral CT. (A) A 76-year-old man with the visualization of AIPDA in a score of 2, suboptimal (main or 1st-order branches). (B) A 46-year-old man with the visualization of PSPDA in a score of 3, moderate (2nd-order branches). (C) A 74-year-old man with the visualization of ASPDA in a score of 4, superior (3rd- order branches). (D) A score of 5, a 61-year-old woman with the visualization of DPA in a score of 5, excellent (4th or higher order branches).

The visualization of the branch order of the pancreatic arteries was assessed with 5-point scale: 5, excellent (4th or higher order branches); 4, superior (3rd order branches); 3, moderate (2nd order branches); 2, suboptimal (main or 1st order branches); 1, poor (vessels not seen)^3^ (Figure 3). The score of the branch order was assessed by 2 radiologists independently and averaged for a mean value.

### Statistical Analysis

A Mann–Whitney *U* test was used to investigate the difference in subjective scores obtained from polychromatic and monochromatic image. An independent *t*-test was performed on the quantitative parameters obtained from polychromatic and monochromatic image. Chi-square test was applied to assess differences in display rate of segment of pancreatic artery between polychromatic and monochromatic image. The interobserver agreement between the 2 radiologists was evaluated using kappa statistics. A kappa value of 0.40 or less indicated fair agreement; 0.41 to 0.60 was moderate; 0.61 to 0.80, substantial agreement; 0.81 or greater, almost perfect agreement. All statistical analyses were done with software SPSS version 17.0 for windows (SPSS Inc, Chicago, IL). All values were presented as mean ± standard deviation. For all comparisons, a *P* value of less than 0.05 was considered significant.

## RESULTS

### Patient Demographics

The weight and BMI of patients between 2 groups were not significantly different (weight: 67.7 ± 12.0 kg, BMI: 24.3 ± 3.4 kg/m^2^ for spectral CT; weight: 65.7 ± 10.0 kg, BMI: 23.9 ± 2.6 kg/m^2^ for conventional CT; *P* = 0.765, 0.610, respectively). The radiation dose was similar between 2 groups (effective dose: 6.7 ± 0.7 mSv for spectral CT vs 6.3 ± 2.1 mSv for conventional CT, *P* = 0.870). The best energy range to display the pancreatic arteries was 53 to 66 keV among these 26 patients.

### Quantitative Analysis for Monochromatic and Polychromatic Images

The CT number of SPA and pancreas, image noise, CNR, SNR, and SIR between 2 groups were described in Table 1. Compared with polychromatic image, the CNR, SIR, and SNR obtained by monochromatic images were increased by 64.74%, 23.99%, and 39.50%, respectively (*P* = 0.000, 0.011, 0.001). There were no statistically significant differences in image noise and CT number of pancreas between 2 groups (*P* *>* 0.05).

**TABLE 1:**
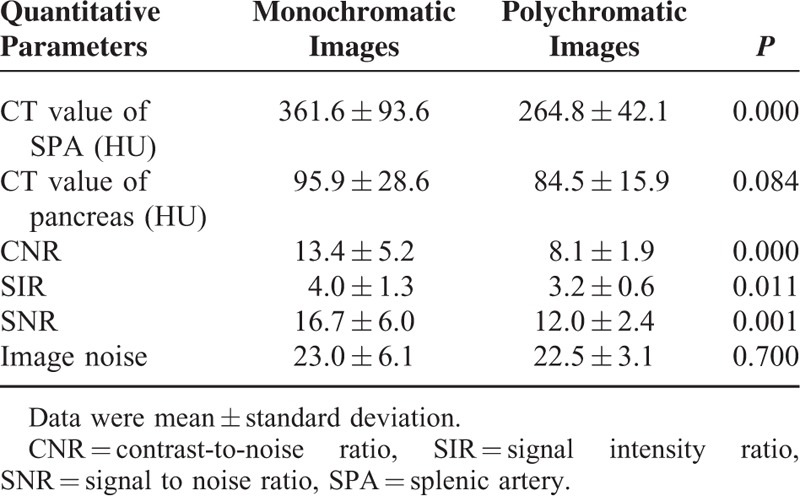
The Quantitative Parameters for Monochromatic and Polychromatic Images

### Score of the Branch Order for Monochromatic and Polychromatic Images

The score of the branch order was significantly higher for PSPDA, ASPDA, and DPA in the monochromatic images than in the polychromatic images (*P* < 0.05) and were slightly better in the monochromatic images for posterior inferior pancreaticoduodenal artery (PIPDA) and anterior inferior pancreaticoduodenal artery (AIPDA) (*P* > 0.05) (Table 2). The kappa value of the independent ratings of branch scores for the 2 independent radiologists were 0.64 for monochromatic images and 0.66 for polychromatic images indicating substantial agreement.

**TABLE 2:**
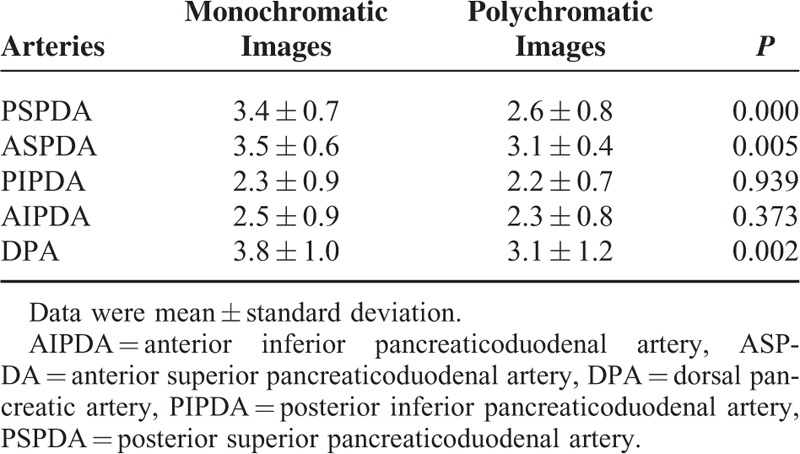
The Scores of Branch Order for Monochromatic and Polychromatic Images

At monochromatic images, the display rates of 2nd order branches in PSPDA, ASPDA, and DPA were 88.46% (23/26), 96.15% (25/26), and 88.46% (23/26), respectively, while the display rates of 3rd order branches in PSPDA, ASPDA, and DPA were 26.92% (7/26), 34.62% (9/26), and 61.54% (16/26).

### Assessment of the Course of Pancreatic Arteries for Monochromatic and Polychromatic Images

The display rate was significantly better in monochromatic images for the 2nd and 3rd segments of PSPDA, the total course of ASPDA and the artery of uncinate process (*P* < 0.05) (Table 3). The kappa value of the independent ratings of assessment of the course of pancreatic arteries for the 2 independent radiologists were 0.89 for monochromatic images and 0.90 for polychromatic images, indicating almost perfect agreement.

**TABLE 3:**
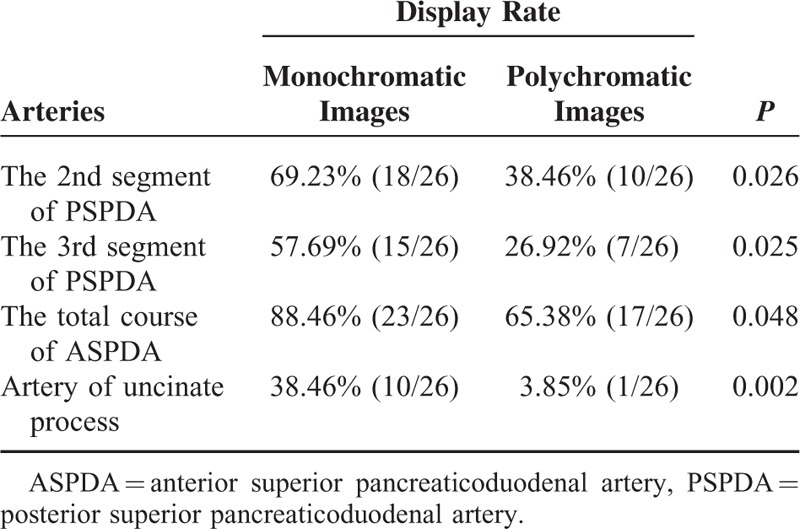
The Course of Pancreatic Arteries for Monochromatic and Polychromatic Images

## DISCUSSION

Previous studies indicated that monochromatic images can yield improvements in the visualization of the enhanced vessels.^3,7–11^ At first, the series of monochromatic images generated from the spectral CT imaging provided the opportunity to select the optimal energy level for depiction of the pancreatic arteries and beam-hardening artifacts could be avoided;^3^ Second, greater image contrast in monochromatic images may be the main reason for the achievement of better demonstration of pancreatic arteries; Third, an adaptive statistical iterative reconstruction (ASIR 30%) was applied for spectral CT to suppress the image noise and improve image quality.^16,17^ All these reasons may contribute to the differences in the visualization of the enhanced vessels between two groups. Furthermore, compared with polychromatic images, spectral CT with monochromatic images can improve the visualization of pancreatic arteries without increasing radiation dose.

Branch visualization of pancreatic arteries was better at monochromatic images than at polychromatic images. The visualization of branch order was best in the DPA among the 5 pancreatic arteries. On the one hand, the caliber of the DPA was larger than other pancreatic arteries. On the other hand, the course of DPA, which was parallel to the long axis of pancreatic body and tail, was more suitable for the visualization of peripheral branches, while the course of other arteries was more tortuous than the course of DPA. In addition, our study showed that the 2nd branches of PSPDA, ASPDA, and DPA were well displayed in monochromatic images and 3rd or higher order branches of DPA were observed in about 61.54% of patients.

Little attention has been paid to the course of the pancreatic arteries at CT. Our study showed a detailed assessment of pancreatic anatomy. The display rate was significantly better in monochromatic images for the 2nd and 3rd segments of PSPDA, the total course of ASPDA, and the blood supply of uncinate process. The relationship between PSPDA and common bile duct was well assessed in monochromatic images. Our results also showed that the ASPDA did not always remain on the anterior surface of pancreatic head; it could go along the posterior surface of the uncinate process where it anastomosed with the AIPDA. No previous reports have mentioned the artery of uncinate process at CT, although it may originate from the truck or right branch of DPA and appear as small linear enhanced structure crossing the superior mesenteric vein posteriorly. In our study, about 38.46% of 26 cases with monochromatic imaging had the artery of uncinate process, while the display rate of it was reported as 60% at anatomic studies.^15^ We supposed that in our study, the artery of uncinate process all originated from the trunk of the DPA but those which originated from the right branch of DPA was not seen due to its smaller diameter.

It could be potentially important to evaluate the anatomy of these pancreatic arteries. Understanding the normal pancreatic arteries was especially important to interventional procedures, such as transarterial chemotherapy and transarterial embolization. In addition, familiarization with the anatomy of pancreatic arteries was a prerequisite for evaluating the blood supply to pancreatic tumors. Furthermore, preoperative displaying the relationship between PSPDA and common bile duct was the essential to avoid the ischemia of the biliary duct in the duodenum-preserving head resection. Preoperative assessment of the artery of uncinate process was helpful to avoid the ischemia and necrosis of uncinate process in the pancreatic body and tail resection.

Our study had several limitations. First, in our study, the pancreatic arteries did not have anatomic reference standard in all cases. Second, there were no lesions of pancreas analyzed. Focused studies on the relationship between pancreatic diseases and pancreatic small vessels with a different study protocol will be needed to be further investigated.

## CONCLUSION

Compared with conventional polychromatic images, monochromatic images can improve the visualization of the branches of pancreatic arteries and increase the display rate of the 2nd and 3rd segments of PSPDA, the total course of ASPDA, and the artery of uncinate process.
